# Enhancing electromagnetic tracking accuracy in medical applications using pre-trained witness sensor distortion models

**DOI:** 10.1007/s11548-023-02994-z

**Published:** 2023-07-27

**Authors:** Marco Cavaliere, Pádraig Cantillon-Murphy

**Affiliations:** 1 University College Cork, Cork, Ireland; 2 Tyndall National Institute, Cork, Ireland

**Keywords:** Electromagnetic tracking, EMT, Distortion, Ultrasound, Fluoroscopy, Witness sentinel sensor

## Abstract

**Purpose:**

Electromagnetic tracking (EMT) accuracy is affected by the presence of surrounding metallic materials. In this work, we propose measuring the magnetic field's variation due to distortion at a witness position to localise the instrument causing distortion based on a pre-trained model and without additional sensors attached to it.

**Methods:**

Two experiments were performed to demonstrate possible applications of the technique proposed. In the first case, the distortion introduced by an ultrasound (US) probe was characterised and subsequently used to track the probe position on a line. In the second application, the measurement was used to estimate the distance of an interventional fluoroscopy C-arm machine and apply the correct compensation model.

**Results:**

Tracking of the US probe using the proposed method was demonstrated with millimetric accuracy. The distortion created by the C-arm caused errors in the order of centimetres, which were reduced to 1.52 mm RMS after compensation.

**Conclusions:**

The distortion profile associated with medical equipment was pre-characterised and used in applications such as object tracking and error compensation map selection. In the current study, the movement was limited to one degree of freedom (1 DOF) and simple analytical functions were used to model the magnetic distortion. Future work will explore advanced AI models to extend the method to 6 DOF tracking using multiple witness sensors.

## Purpose

Electromagnetic tracking (EMT) is used in medical applications to localise targets without a line of sight, such as during image-guided interventions or in situations where the target is hidden behind tissue or other obstructions [1, 2]. One of the main technical challenges in using EMT is the low accuracy achieved in the presence of surrounding metallic materials, which can distort the magnetic field and cause errors in the measurements [3–5].

Offline volume characterisation is a solution for static distortion scenarios [6]. However, in the varying environment of the operative room, the dynamic distortion effect is unknown and cannot be corrected by pre-operative calibration.

In this article, we propose to use witness sensors measuring the deviation of the magnetic field due to distortion caused by medical equipment. By pre-training a model on different distortion configurations, the aim is to predict the position and orientation of the instrument based on the measured distortion.

The idea of distortion detection by monitoring the field variation at known positions has been proposed in the literature [7, 8]. However, using that information to localise the instrument causing distortion has never been investigated with experimental methods.

A second compensation approach is proposed where static compensation maps, recorded for pre-determined distortion configurations, are interpolated to obtain a new map that applies to an unseen distortion scenario. An external optical tracking system might be employed to identify the correct interpolation plane [9]. Alternatively, following the method proposed in this article, a witness sensor was used to estimate the correct compensation map without additional tracking systems.

In this work, we present a novel technique to estimate the position of medical instruments based on their distortion effect on a witness sensor at a fixed position. The method is then demonstrated for one-degree-of-freedom (1 DOF) applications. Future improvements to the current solution are also discussed.

## Methods

The open-source electromagnetic tracking system Anser EMT [10] was used because it allowed access to the raw field measurements. Two experiments were performed to demonstrate possible applications of the technique proposed in this article.

**Fig. 1:**
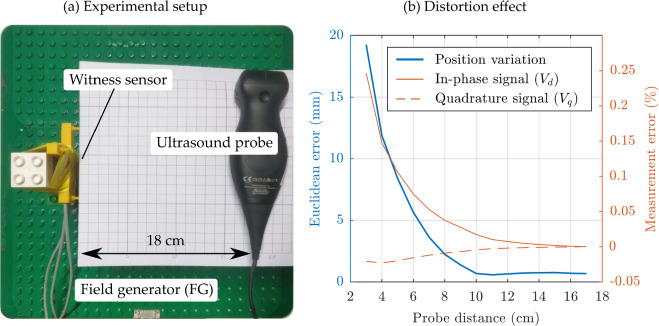
Experiment A. **a** US probe was moved on a line approximately 15 cm above the field generator, and a witness sensor at a fixed position recorded the magnetic field variation. **b** Magnetic distortion affects the EMT position error, calculated as the Euclidean distance in millimetres, and the direct and quadrature components of the field measurement, reported as the percentage deviation from the undistorted value

In the first experiment, a magnetic sensor (3DV11AOI-A-S0600J, Grupo Premo, Malaga, Spain) was placed approximately 15 cm above the planar field generator (FG), and a commercially available Ultrasound (US) probe (Butterfly iQ + , Butterfly Network, Burlington, MA, US) was moved on a line in steps of 1 cm, as shown in Fig. 1a. The distortion effect on the EMT position of the sensor and the associated magnetic measurements were recorded. The distortion variation is visualised in Fig. 1b.

The magnetic field of the EMT system used is modulated at a frequency between 2 and 3 kHz. Therefore, the distortion was mainly caused by eddy currents induced in the conductive distorter [11], as demonstrated by the appearance of an out-of-phase, or quadrature, component (${V}_{q}$) in the received signal, which is evident in Fig. 1b. The ${V}_{q}$ sinusoidal signal component is 90 degrees delayed relative to the in-phase, or direct, component (${V}_{d}$) generated by the EMT system and is caused by the magnetic field that originates from eddy currents.

The distance of the US probe, $D$, was modelled as a function of ${V}_{d}$ and ${V}_{q}$. A polynomial function was empirically found to accurately model the relation between $D$ and the distorted signal while avoiding overfitting:

1 $$D={p}_{0}+{{p}_{d1}{V}_{d}+{p}_{d2}{V}_{d}^{2}+p}_{q1}{V}_{q}+{p}_{q2}{V}_{q}^{2}$$

where the coefficients ${p}_{i}$ must be obtained to fit real data measurements. It should be noted that other metallic objects might cause a different distortion profile. Therefore, lower or higher-order polynomials might be included in Eq. (1) to model the distortion effect.

The proposed technique involves extracting the ${V}_{d}$ and ${V}_{q}$ signal components from a new field measurement of the witness sensor, then using Eq. (1) to determine $D$ and, in this way, estimate the probe's position on the line.

In the second experiment, a cone beam computed tomography (CBCT) scanner (Artis zeego, Siemens Healthineers, Germany) was used in combination with the Anser EMT system to simulate a hybrid EMT and X-ray navigation framework [12]. The proximity of the interventional C-arm caused significant metallic distortion that led to the degradation of the EMT accuracy.

**Fig. 2:**
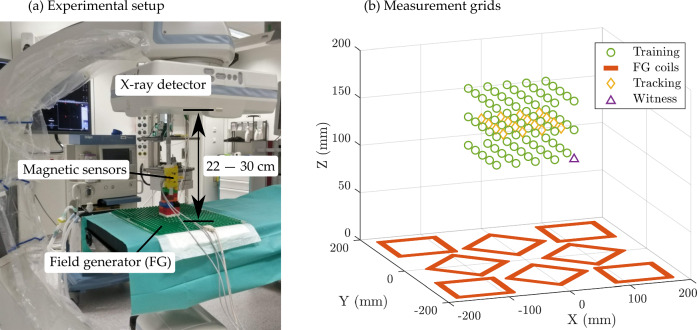
Experiment B. **a** X-ray detector was moved at different distances from the field generator (22, 24, 26, and 30 cm), and the magnetic field vector was captured on the training grid. The X-ray detector was then placed at 28 cm, the distance was estimated from the witness sensor measurement, a new calibrated field model was calculated based on the values pre-characterised, and the EMT error was evaluated on the tracking grid. **b** Position of the training grid, the tracking grid, and the witness sensor relative to the field generator coils

The distorted magnetic field was pre-characterised within the tracking volume by moving magnetic sensors on a grid of $5\times 5\times 3$ training points, where the magnetic field vector was captured. The characterisation procedure was repeated for different distances of the X-ray detector above the field generator (22, 24, 26, and 30 cm), and Duplo blocks (The Lego Company, Billund, Denmark) were used to provide accurate and consistent support. The experimental setup is shown in Fig. 2.

The X-ray detector was then moved to a height of 28 cm, generating a new distortion scenario not previously characterised. The technique presented in this work was used to estimate the unseen distorter's position from the witness sensor measurement. A compensated magnetic field model was obtained using a second-degree polynomial fit of the tabulated characterisation maps, with cubic spline interpolation between the grid points.

The EMT accuracy of the system was evaluated on a grid of $4\times 4$ test points, comparing both the distorted and the compensated magnetic models to solve for the sensor pose. It should be noted that, in this case, the sensors were tracked using traditional EMT methods. The witness sensor was solely used to estimate the X-ray detector location without requiring an external tracking system and, in this way, enabled real-time compensation of the dynamic distortion using a single EMT system.

## Results

**Fig. 3:**
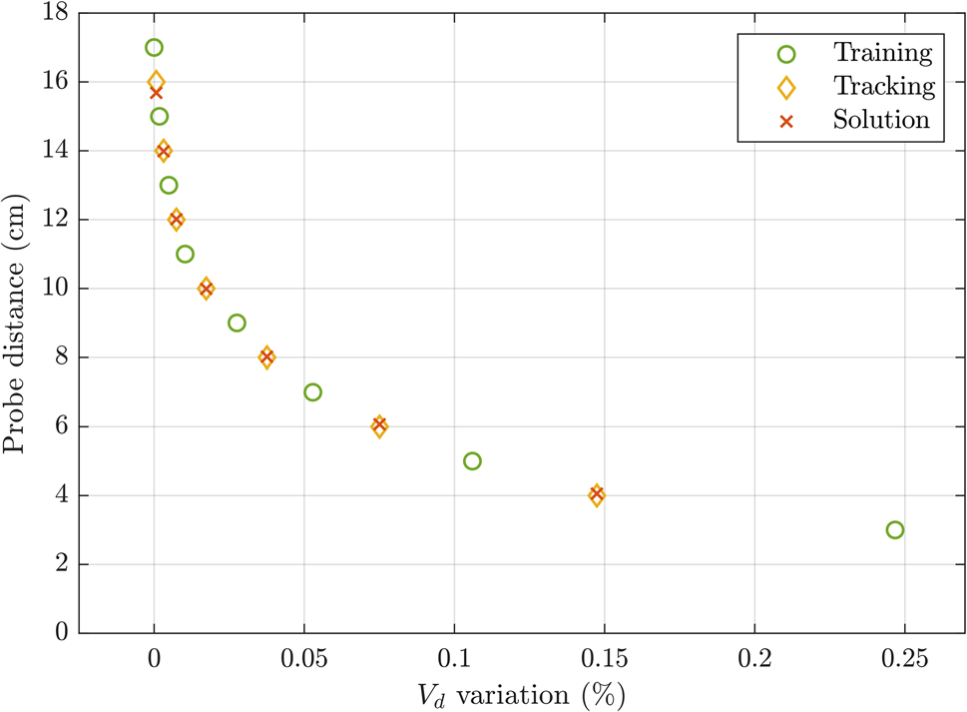
Distortion model. Distortion-based tracking with one degree of freedom as a function of the magnetic measurement variation due to distortion. Note that the plot shows the dependence on ${V}_{d}$, but also ${V}_{q}$ is used to estimate the distance, $D$, as per the distortion model outlined in Eq. (1)

The effectiveness of the distortion model outlined in Eq. (1) is demonstrated in Fig. 3. The polynomial coefficients of Eq. (1) were fitted using every second point with a spacing of 2 cm (training points), whereas the distortion model was tested on the points in between (tracking points), as seen in Fig. 3.

The results of the 1 DOF tracking of the US probe are presented in Fig. 3. The root-mean-square error (RMSE) calculated over the seven test points is 1.25 mm. The maximum error (MAXE) of 3.16 mm was obtained with the instrument placed at the highest distance of 16 cm, when the distortion effect and, consequently, the information captured by the witness sensor were minimal.

For the experiment aimed to compensate for the distortion introduced by the fluoroscopy machine, the distance of the X-ray detector was predicted as 28.1 cm, based on the witness sensor measurement. Considering that the reference distance of 28 cm was known within 0.5 cm tolerance, this might also account for the 1 mm error.

The static tracking test was carried out for Y (horizontal) and Z (vertical) orientations of the magnetic sensor, in the presence of the distorter at 28 cm, both before and after using the distortion compensation method described above. The results obtained in a distortion-clean environment were also included for comparison.

Position errors were calculated as the Euclidean distance between the EMT solution and the reference defined by the Duplo grid. The orientation errors were calculated as the absolute angular difference. Error statistics are summarised in Table 1, where the root-mean-square (RMSE), the 50th percentile (PRC50), and the maximum (MAXE) errors are reported.

**Table 1:** EMT error statistics

Position/angle	Without distortion		With distortion		Distortion compensation	
	Y	Z	Y	Z	Y	Z
RMSE (mm/deg)	2.15/0.94	2.89/1.48	2.93/1.38	11.45/9.35	1.36/0.48	1.66/0.92
PRC50 (mm/deg)	1.78/0.87	2.6/1.22	2.42/1.24	11.62/10.24	1.27/0.35	1.38/0.78
MAXE (mm/deg)	3.8/1.42	5.46/2.96	5.84/2.28	16.23/12.5	2.58/1.04	3.54/1.88

Position and orientation EMT errors of Y (grey shading) and Z-oriented sensors in different distortion scenarios

**Fig. 4:**
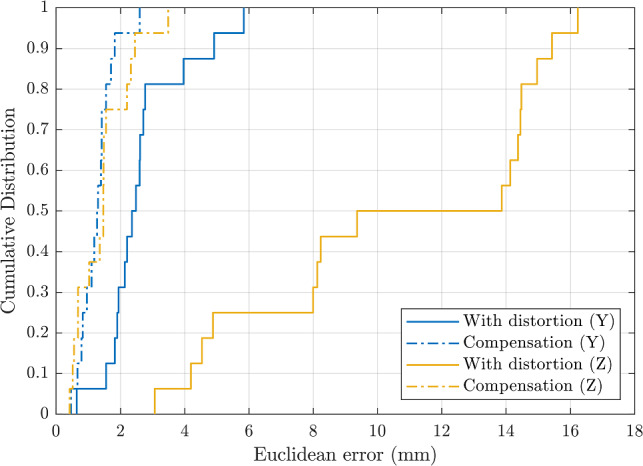
Cumulative error. Cumulative distribution of the position error with the X-ray detector placed 28 cm above the field generator for Y and Z orientations of the sensor. Errors are shown before and after applying the compensated magnetic field model to solve for the sensor position

The cumulative position EMT error is visualised in Fig. 4, which demonstrates how the error distribution changed for the two sensor orientations after applying the compensated magnetic model.

## Conclusions

While magnetic distortion is a limitation for EMT, it can also be viewed as an effect to leverage. In this article, we presented the idea of distortion tracking using a witness sensor placed at a fixed position and performed experimental investigations as proof of concept.

The US probe and the fluoroscopy C-arm were successfully localised with 1 mm accuracy using the proposed technique. Moreover, knowledge of the C-arm position allowed us to interpolate between previously collected magnetic field maps and apply the updated field model to compensate for the unseen distortion scenario.

The X-ray detector above the tracking volume introduced significant EMT errors, mainly affecting the sensor oriented along the Z-axis (vertical) of the planar field generator. In this case, the distortion caused an RMSE increment from 2.9 to 11.5 mm, which was reduced to 1.7 mm after compensation. The orientation error followed a similar pattern, increasing from 1.5° to 9.5° and being reduced below 1° after correction.

The accuracy requirements vary depending on the specific procedure. However, it is generally agreed that tracking errors at millimetre and degree levels are sufficient for most endoscopic and surgical navigation tasks [13–15].

The current study was limited to one witness sensor and 1 DOF tracking, but the method can be quickly extended to multiple dimensions if additional witness sensors are included. The main challenge will be the accurate modelling of varied distortion configurations to take into account how the distortion is captured by multiple sensors when the distorter is placed at different positions and orientations. In this case, the simple analytical model used in this work, presented in Eq. (1), might not be sufficient and more advanced methods should be investigated. Even so, the technique will require specific pre-characterisation and training for every instrument to be tracked, and simultaneous tracking of multiple targets might not be feasible.

Future work will explore optimised topologies of multiple witness sensors, deep learning methods for modelling magnetic distortion, and alternative electromagnetic field generation techniques to increase the distortion effect artificially. In this way, we plan to extend the current method and achieve accurate 6 DOF tracking of medical instrumentation without the requirement of a target sensor attached to them.
